# A proteomics investigation of primary human articular chondrocyte isolation

**DOI:** 10.1016/j.ocarto.2025.100664

**Published:** 2025-08-23

**Authors:** Abby Brumwell, Simran Raheja, William Cawley, Emily Birkett-Jones, Sarah E. Orr, Caitlin Todd, David J. Deehan, Nichola J. Conlon, Matthias Trost, Sarah J. Rice

**Affiliations:** a Skeletal Research Group, Newcastle University, International Centre for Life, Newcastle Upon Tyne, NE3 1UP, UK; b Biosciences Institute, Newcastle University, Framlington Place, Newcastle Upon Tyne, NE2 4HH, UK; c Newcastle University Teaching Hospitals NHS Trust, Freeman Hospital, High Heaton, NE1 7DN, UK; d Nuchido Ltd., Dissington Hall, Dalton, Northumberland, NE18 0AD, UK

**Keywords:** Collagenase, Osteoarthritis, Chondrocytes, Phenotype, Proteomic, Transcriptomic

## Abstract

**Objective:**

Human primary articular chondrocytes (hPACs) are routinely isolated from articular cartilage for pre-clinical OA research. Collagenase digest of tissue is an essential step, yet the impact of lengthy enzymatic incubation on the hPAC phenotype is unclear. We aimed to delineate this through proteomic analysis.

**Design:**

hPACs were isolated from human knee cartilage (n ​= ​4) from patients undergoing total knee replacement. Collagenase treatment was performed with or without prior fixation of the tissue. Proteomes were quantified using LC-MS/MS. The Proteomic Ruler was employed to estimate protein copy numbers and cell protein masses. Significant differences in protein intensities were determined using paired t-testing and Benjamini-Hochberg correction. Proteomic data were integrated with existing transcriptomes (GSE217871) of hPACs and ground cartilage (*ex vivo*) RNA.

**Results:**

Following collagenase treatment, we identified 498 differentially expressed proteins (DEP) in the unfixed cells. We observed depletion of FOXO signaling and enrichment of ribosomal RNA processing, indicative of increased cell cycle progression. This was supported by depletion of cell cycle inhibitors including CDKN1C. Transcriptomic analysis identified 3937 differentially expressed genes (DEG), and a 53 ​% overlap in DEP and DEG. Propidium iodide staining did not identify significant differences in cell cycle between fixed and unfixed hPACs.

**Conclusions:**

We identified shifts in the proteome and transcriptome of hPACs following collagenase digest, supporting the use of tissue fixation before extracting nucleic acids for analysis where possible. Despite widespread expression changes, hPACs largely retain their chondrocyte phenotype. These datasets and analyses will serve as a valuable resource for the OA and cartilage research community.

## Graphical abstract

**Figure:**
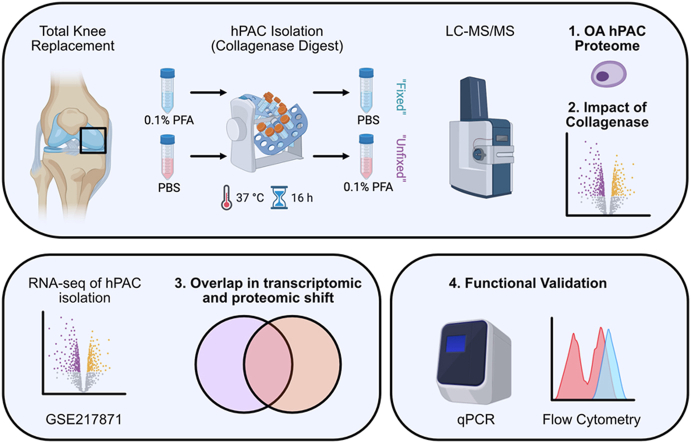

## 1 Introduction

Cartilage consists of a single cell type: the chondrocyte. This specialized tissue is avascular and, consequently, chondrocytes are adapted to thrive in hypoxic environments, taking responsive cues from exogenous factors including oxygen concentration, mechanical forces, and matrix composition. Articular cartilage is also hypocellular, with chondrocytes occupying just 1–2 ​% of the total volume. Post-development, articular chondrocytes are non-proliferative yet remain metabolically active. Their primary role is to maintain the balance of anabolism and catabolism within the extracellular matrix. The specialized load bearing and compressive properties of cartilage are possible due to the unique structure of the dense extracellular matrix and pericellular matrix encasing the cells.

Osteoarthritis (OA) is a degenerative disease hallmarked by the breakdown of articular cartilage. This leads to a loss of joint function, chronic pain and disability. The dearth of disease-modifying therapies for OA leads to a common clinical end point of joint replacement surgery (arthroplasty) in late-stage disease. Over 200,000 hip and knee arthroplasty surgeries are conducted annually in the UK alone, as a direct result of OA [1]. Consequently, human joint tissues, including cartilage, bone, and synovium, are often readily accessible for analysis by researchers investigating the molecular drivers of OA.

The matrix-dense composition of skeletal tissues presents unique technical hurdles to isolating distinct cellular components [2]. The application of novel sequencing technologies in skeletal tissues is frequently overlooked by researchers constructing pan-tissue atlases using novel methodologies. Even within laboratories which specialize in the handling and dissociation of such tissues, the adaptation of protocols for matrix-dense and mineralized tissues can prove challenging.

The use of human primary articular chondrocytes (hPACs) in culture requires a lengthy (often overnight) collagenase digest of cartilage [[3], [4], [5], [6]]. This subjects the cells to exogenous stresses, typically in normoxic environments, likely leading to a phenotypic shift. Chondrocyte isolation methods vary in both the types of collagenase used, the concentration, and time over which digest occurs [7]. Several published recommendations have been made in attempts to standardize the field, and increasingly a short pre-digest of cartilage with pronase (0.1–0.4 ​%) is being adopted to increase the efficacy of collagenase [3,8,9]. Despite this, protocols vary between laboratories [10]. In 2022, Shen and colleagues*,* optimized collagenase digest of human primary articular chondrocytes (hPACs) from articular cartilage to preserve the transcriptome for downstream RNA-sequencing experiments. They compared the global transcriptomic profile of ground cartilage (*ex vivo*) to a range of collagenase digest treatments (4–18h) with and without Actinomycin D to block RNA transcription. They demonstrated that the addition of Actinomycin D, and a shorter (4h) incubation period with collagenase, preserved the chondrocyte transcriptome most closely with ground tissue, based on principal component analysis and the expression of 13 preselected gene markers [11].

Increasingly, for modern omics protocols, where possible, tissue samples are fixed prior to nuclear dissociation or single cell analyses. Yet, for those seeking to isolate hPACs for downstream *in vitro* culture, this is unfeasible. Despite the quiescent state of chondrocytes within the cartilage matrix, researchers have been isolating hPACs from arthroplasty joints for over 50 years, observing mitotic proliferation of cells in 2D culture [12,13]. It is well established that normoxic, 2D monolayer culture of chondrocytes results in dedifferentiation over multiple passages, measured through fibroblastic morphology, reduced matrix production, and altered gene expression profiles [7,13,14]. Mimicking physiological conditions such as hypoxic environments (1 ​% O_2_) [14], culture in pro-chondrogenic growth medium [15], or 3D culture [16], can help to maintain or restore the chondrocyte phenotype. To our knowledge, no published studies have yet reported how the hPAC phenotype shifts during the collagenase isolation process at a molecular level.

In this study, we conducted proteomic analysis of hPACs with fixation prior to or post enzymatic digest. We will herein refer to these conditions as “fixed” and “unfixed” hPACs, respectively. Using data from the fixed hPACs, we first present the proteomic fingerprint of the human OA knee chondrocyte using the “Proteomic Ruler” to estimate the copies of intracellular proteins. We then identified significant changes in the proteome following cartilage digest with or without prior fixation. Using available RNA-seq data, we also conducted differential gene expression analysis and compared the transcriptomic changes with those observed in the proteome. Finally, we validated the observed changes using qPCR and flow cytometry in an independent cohort of patient samples.

## 2 Methods

### 2.1 Sample collection and processing

Human knee cartilage was obtained with informed consent from patients undergoing elective total joint replacement surgery for primary knee OA. Ethical approval was granted by the Newcastle and North Tyneside Research Ethics Committee (REC reference number 19/LO/0389). Full details of all patient samples used in this study are presented in Supplementary Table 1.

Macroscopically intact cartilage was dissected from the femoral condyles and tibial plateau. For each sample, cartilage fragments (1.5g) were separated for collagenase digestion with (“fixed”) or without (“unfixed”) prior fixation (0.1 ​% PFA, 2h, 4 ​°C). The tissue was washed in PBS and pre-digested in 0.1 ​% Pronase (Roche; 1h, 37 ​°C) before overnight digestion in 0.1 ​% Type I collagenase (Sigma Aldrich) at 37 ​°C with mechanical agitation. All enzymatic steps were conducted in DMEM (+4.5 ​g/L d-glucose, l-glutamine, sodium pyruvate) (Gibco) supplemented with 10 ​% FBS (Gibco), 200 U/ml penicillin-streptomycin (Gibco) and 40U/ml Nystatin (Sigma-Alrich). Resultant cell suspensions were passed through a 100 ​μm cell strainer, washed in PBS then centrifuged again (1200 ​rpm, 5 ​min). Unfixed samples were fixed in PFA post-digestion and washed twice in PBS. Cells were then centrifuged to removed PBS, and the pellets were snap frozen in liquid nitrogen and stored at −80 ​°C.

### 2.2 Protein peptide injection and Liquid chromatography (LC)

Cell pellets were resuspended in lysis buffer (5 ​% SDS, 50 ​mM triethyl ammonium bicarbonate, protease inhibitors (cOmplete, Roche) and DNase (universal nuclease for cell lysis, Pierce)) to extract whole cell protein lysates. 50 ​μg of protein was reduced (20 ​mM tris-(2-carboxyethyl)phosphine (TCEP)), alkylated (20 ​mM iodoacetamide) and acidified (27.5 ​% phosphoric acid) then prepped using S-trap micro columns (Protifi). On-column protein digestion was performed using trypsin (1:10 w/w) for 2 ​h at 47 ​°C. Peptides were eluted (50 ​mM TEAB, 0.2 ​% formic acid and 50 ​% acetonitrile) then dried.

Eluted protein peptides were resuspended in 0.1 ​% formic acid in water and loaded onto C18 Evotips (Evosep, Odense, Denmark) following manufacturer recommendations.

### 2.3 Data- independent acquisition LC-MS/MS

LC was performed using an Evosep One system with a 15 ​cm Aurora Elite C18 column with integrated captivespray emitter (IonOpticks), at 50 ​°C. Immediately prior to LC-MS, peptides were resuspended in buffer A (0.1 ​% formic acid in HPLC water) and a volume of peptides equivalent to 500 ​ng was loaded onto the LC system-specific C18 EvoTips and subjected to the predefined WhisperZoom 20 SPD protocol (where the gradient is 0–35 ​% buffer B (0.1 ​% formic acid in acetonitrile) at 200 ​nl/min, for 58 ​min. Data-independent acquisition parallel accumulation with serial fragmentation was selected as the operating mode for the timsTOF-HT. TIMS ion accumulation and ramp times were set to 100 ​ms, and total cycle time was ∼1.8 ​s, with mass spectra acquiring between 300 and 1200 ​*m*/*z*. The instrument was operated in Data-independent acquisition parallel accumulation with serial fragmentation mode using variable width IM-m/z windows, as optimized by py_diAID [17]. Collision energy was set to occur in a linear fashion, from 20 ​eV to 59 ​eV with ion mobility of 0.6–1.6 ​V ​s/cm^2^.

### 2.4 MS data analysis

DIA files were analysed library-free using DIA-NN (version 1.9), and searched against the SwissProt *Homo sapiens* database (20589 sequences; downloaded August 15, 2023) and contaminant FASTA file [17]. The following search parameters were applied in DIA-NN: respective minimum and maximum peptide lengths of 7 and 30 ​amino acids, a precursor charge range of 1–4, a precursor *m*/*z* range of 300–1200, and a fragment ion *m*/*z* range of 200–1800. Cysteine carbamidomethylation was enabled as a fixed modification, and N- terminal M excision as a variable modification. Trypsin specificity was set to one missed cleavage and a protein PSM false discovery rate of 1 ​%. Non-normalized intensity data was log_2_ transformed and filtered to 1) exclude contaminants, 2) contain at least two unique peptides, and 3) for at least 70 ​% valid values in at least one group. 7009 proteins were retained for downstream statistical analysis, i.e., paired t-tests with Benjamini-Hochberg (BH) correction with a significance threshold of fold change (FC) ​> ​1.5 (log_2_FC ​> ​0.58) and an adjusted *P*-value <0.05, conducted using the R package limma [18]. For estimation of protein copy numbers and cellular masses, the “Proteomic Ruler” was applied as previously described [19].

PathDIP 5 was used to perform curated pathway analyses using KEGG, REACTOME, PathBank, and Panther Pathways on the significantly increased and decreased proteins in the unfixed chondrocytes [20]. STRING analysis was conducted to identify protein networks [21]. Interactions were determined with medium confidence based on experimental and database evidence.

### 2.5 RNA-sequencing analysis

Transcriptomic data of RNA extracted from either ground cartilage (*ex vivo*) or from isolated hPACs (18h collagenase digest) was downloaded from the NIH Gene Expression Omnibus (GEO; GSE217871) [11]. Paired differential expression analysis was carried out using the DESeq2 package [22] in R. The DESeq2 data underwent a diagnostic dispersion estimate to model biological noise (Fig. S3), before undergoing a regularised logarithmic transformation. The Euclidean distance between sample groups was calculated to test for outliers (Fig. S4). Transcripts were defined as differentially expressed when the FC was >2 (log_2_FC ​> ​1.0) and the BH-adjusted *P-*value (Padj.) <0.05. The RNA-seq data was annotated with the UniProt ID of the encoded protein using the biomaRt package [23] and overlapped with proteomics data.

To calculate transcripts per million for visualization of RNA-seq data, Reads Per Kilobase (RPK) were calculated by dividing average read counts per sample by gene length. Reads Per Kilobase values were then normalized by dividing by the sum of all Reads Per Kilobase values and multiplying by 10^6^. Quality control was performed by visualizing the transcripts per million distribution and correlating the TPMs between samples to test for clustering by experimental condition (Figs. S5 and S6).

### 2.6 Gene expression analysis

Nucleic acids were extracted from frozen ground knee cartilage tissue by separation with TRIzol chloroform (Life Technologies), followed by RNA purification using the RNeasy Mini Kit (Qiagen), as previously described [24]. RNA (600 ​ng) was treated with DNAse and reverse transcribed using SuperScript VILO cDNA synthesis kit (Invitrogen).

Gene expression was measured by reverse transcription quantitative PCR (RT-qPCR) using pre-designed TaqMAN assays (IDT; Table S2). Each gene was measured in triplicate (technical replicates) within each biological sample. The expression of target genes was normalized to the mean of the housekeeping genes *GAPDH, HPRT1* and *18S* using the method of 2^−Δct^.

### 2.7 Cell cycle detection and analysis

Cells were fixed in ice-cold 70 ​% ethanol for 30min at 4 ​°C then resuspended in PBS containing RNase A (50 ​μg/ml) (Thermo Scientific) and Propidium Iodide (50 ​μg/ml) (Sigma-Aldrich) for 45min at room temperature in the dark (n ​= ​3). Cells were analysed on BD LSRFortessa (BD Biosciences), using a 561 ​nm laser with a 610/20 bandpass filter. Analysis was performed in FCS Express with doublets and debris being excluded based on FSC-H vs FSC-A gating. Cell cycle analysis was performed using the methods of Dean-Jett-Fox [25,26] within the Multicycle DNA tool as previously described [27]. For each sample 5000 ​cells were acquired and analysed.

## 3 Results

### 3.1 The proteomic fingerprint of human articular chondrocytes

**Fig. 1:**
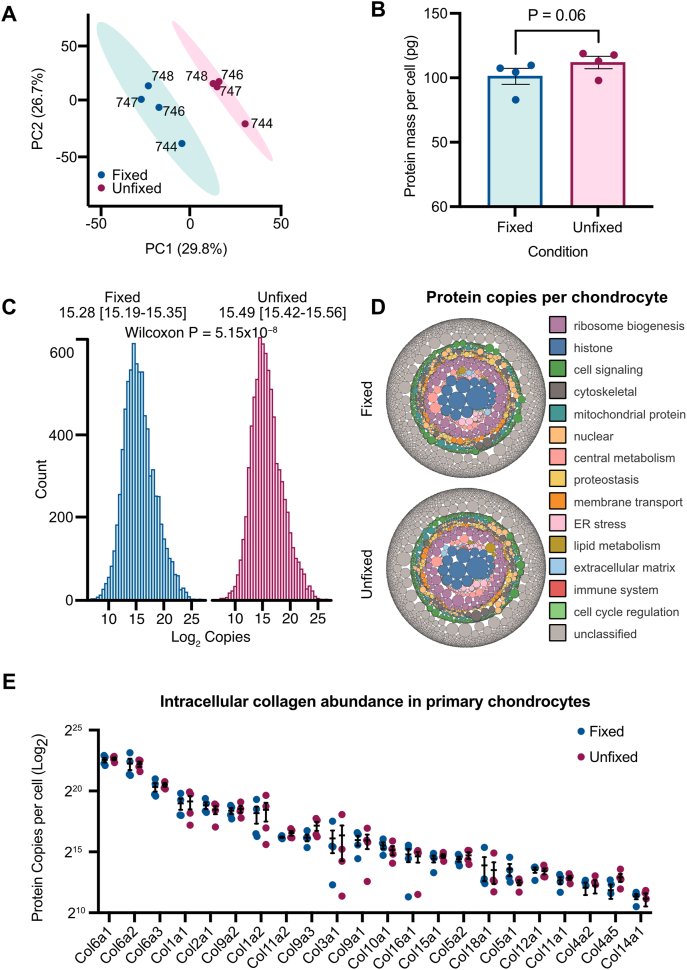
**A quantitative overview of the human primary chondrocyte proteome. A,** PCA plot of fixed (blue) and unfixed (pink) chondrocytes (both n ​= ​4). **B**, Protein mass per cell between the two groups. P-value was calculated using a paired *t*-test. **C,** Average copy number histogram for the chondrocytes. Statistical significance was determined using a Wilcoxon rank sum test with bootstrapping to calculate 95 ​% confidence intervals. **D,** Bubble plot showing proteins colored by specific categories with the size representing the average estimated protein copies. **E,** Collagen protein copies in the fixed and unfixed chondrocytes. (For interpretation of the references to color in this figure legend, the reader is referred to the Web version of this article.)

To establish whether enzymatic isolation of human primary articular chondrocytes (hPACs) changes their phenotype, we first investigated global changes in the hPAC proteome following collagenase digest of cartilage. Principal component analysis revealed clustering of the chondrocyte samples by fixation status (Fig. 1A). Using data from all 7924 proteins detected, the Proteomic Ruler [19] was applied to estimate the copy number and amount of each protein (pg) per cell (Table S3). The average protein mass of the fixed hPACs was 101.5 ​pg (±6.2) per cell compared to 112.2 ​pg (±4.8) per cell in the unfixed hPACs, representing a 10.5 ​% increase (*P* ​= ​0.06; Fig. 1B). This was supported by an increase in the median estimated protein copies per cell from 15.28 [95 ​% confidence intervals 15.19–15.35] to 15.49 [15.42–15.56] following collagenase treatment (P ​= ​5.15 ​× ​10^−8^; Fig. 1C).

The identified proteins were assigned to one of 14 biological categories (3661; 47 ​%), or as “unclassified” based on their UniProt descriptors (Tables S3 and S4). Together, the 14 categories represented 51 ​% of the total protein copies within the cells (Fig. 1D). The summed protein copies per cell were consistently higher in each of the protein categories in the unfixed chondrocytes post-collagenase treatment (Fig. S7).

Collagens account for 60–70 ​% of the dry weight of cartilage [28]. In our study, twenty-four collagens were detected in the chondrocytes, with the most abundant being Col6a1 (6.4 ​× ​10^6^ estimated copies per cell; 95 ​% confidence intervals [5.3–7.5 ​× ​10^6^]) Col6a2 (4.9 ​× ​10^6^; [3.0–6.8 ​× ​10^6^]) and Col6a3 (1.4 ​× ​10^6^; [1.0–1.8 ​× ​10^6^]), the main collagen constituents of the pericellular matrix. Col2a1, the predominant collagen constituent of articular cartilage ECM, was also abundantly expressed with an average of 4.1 ​× ​10^5^ [2.7–5.5 ​× ​10^6^] copies per cell (Fig. 1E).

### 3.2 FOXO signaling is dampened in collagenase-treated chondrocytes

**Fig. 2:**
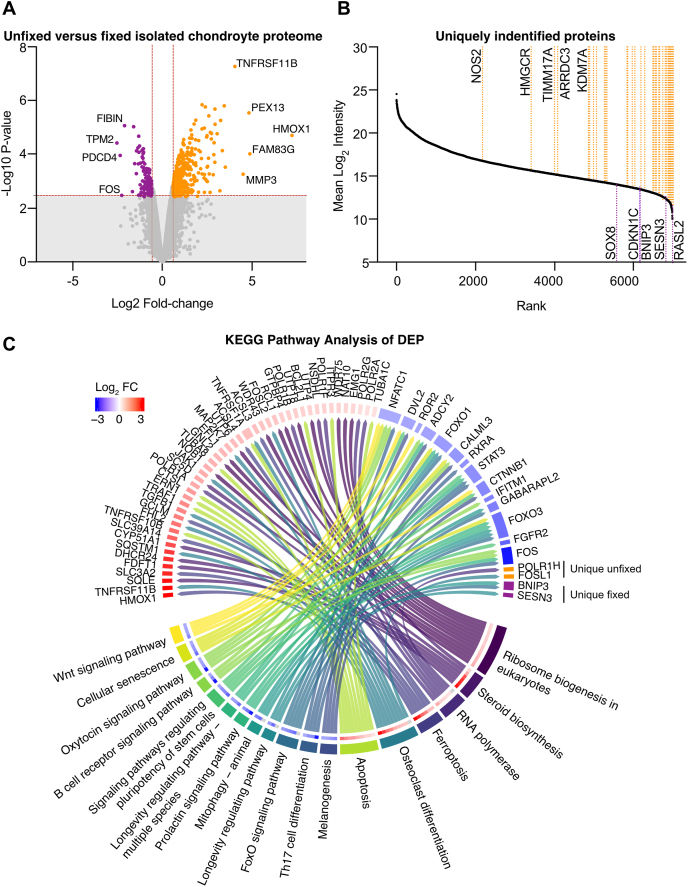
**Differential protein expression analysis between fixed and unfixed human chondrocytes, post-chondrogenesis. A,** Volcano plot showing the proteins which significantly increased (orange) or decreased (purple) in the unfixed chondrocytes following overnight collagenase digest. **B,** Plot of the identified proteins by their mean intensity ranking. Uniquely identified proteins are labelled in purple (unique to fixed) or orange (unique to unfixed). **C,** Chord plot displaying the significant results (FDR <0.05) from the KEGG pathway analysis. (For interpretation of the references to color in this figure legend, the reader is referred to the Web version of this article.)

Proteomes were filtered for paired testing of differential expression between fixed and unfixed hPACs as described in the methods. We identified 7009 proteins which were either detected in at least three sample pairs, or which were uniquely detected in one condition (Table S5). In the unfixed hPACs, 375 proteins significantly increased (Padj. ​= ​4.5 ​× ​10^−4^ - 0.049; log_2_FC ​= ​0.58–7.16) and 123 decreased (Padj. ​= ​0.006–0.049; log_2_FC ​= ​−0.59 to −2.52; Fig. 2A). A further five proteins were uniquely identified in fixed hPACs, and 38 were unique to the unfixed (Fig. 2B–Table S5).

A curated pathway analysis identified 45 pathways amongst the enriched proteins (FDR ​= ​0.049–2.97 ​× ​10^−11^; Table S6) and 47 pathways in the depleted proteins (FDR ​= ​0.049–0.021; Table S7). The enriched KEGG terms, and the proteins comprising these pathways are displayed in Fig. 2C.

**Fig. 3:**
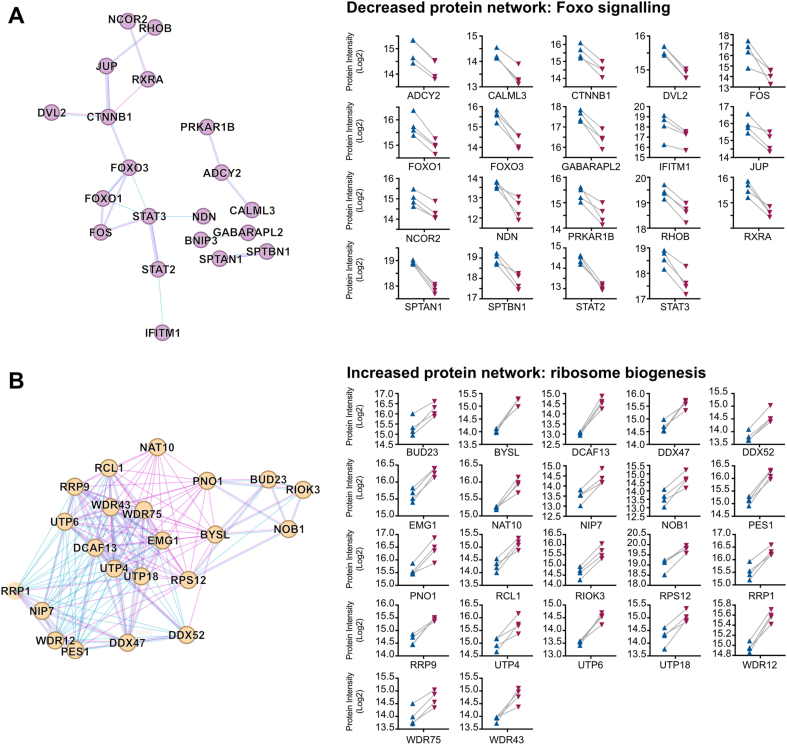
**Protein networks shift during chondrocyte collagenase treatment. A,** STRING network of DEP mapping to Foxo signaling pathways in our PathDIP curated pathway analysis. Nodes are colored purple to represent significantly decreased proteins in unfixed chondrocytes. Edges are colored by the evidence of connections. Pink, experimentally determined; blue, from curated databases. The protein intensities in the fixed and unfixed chondrocytes are displayed on the right-hand side of the panel. **B,** STRING network of 22 DEP mapping to ribosome biogenesis pathways in our PathDIP curated pathway analysis. Nodes are colored orange to represent significantly increased proteins in unfixed chondrocytes. Edges are colored as described in (A). (For interpretation of the references to color in this figure legend, the reader is referred to the Web version of this article.)

The depleted pathways related to FOXO signaling (six terms, FDR ​= ​0.049–0.024), cell senescence and mitophagy (six terms; FDR ​= ​0.046–0.036), and signal transduction terms including Wnt, EGF, and PKA signaling (FDR ​= ​0.039–0.025; Table S7, Fig. 2C). Foxo1 and Foxo3 are two of the four human Foxo family members (and the only two detected in our human chondrocyte samples). They have known roles in regulating the cell cycle and apoptosis and are activated in hypoxic conditions [29]. Here, we observed a significant decrease in expression of Foxo1 and Foxo3 in the unfixed chondrocytes. Foxo1 and Foxo3 individually mapped to 43 pathways, respectively (Table S7), along with 38 additional significantly depleted or uniquely absent proteins in the unfixed chondrocytes. String analysis of these 40 proteins revealed a network of 20 connected nodes (Fig. 3A). BNIP3 (BCL2/adenovirus E1B 19 ​kDa protein-interacting protein 3) was uniquely expressed in the fixed chondrocytes (Fig. 1B), as was CDKN1C (Cyclin-dependent kinase inhibitor 1C), which plays an important role in inhibition of the cell cycle.

The enriched pathways included 45 significant terms (FDR<0.05; Table S6). Of these, seven related to ribosomal RNA processing and ribosome biogenesis (FDR ​= ​0.025–2.9 ​× ​10^−11^), twelve to steroid metabolism and cholesterol biosynthesis (FDR ​= ​0.003–2.9 ​× ​10^−7^), and nine to interferon/interleukin signaling (FDR ​= ​0.044–4.1 ​× ​10^−4^).

Of the 42 proteins mapping to the ribosomal RNA and ribosomal terms, STRING analysis identified a network of 38 (Fig. S8), a subset of which is displayed in Fig. 3B. This includes the UTP proteins 4, 6, and 18, which form part of the UtpA and UtpB processome subunits, initiators of eukaryotic ribosome assembly [30]. Additionally, the network contains three members of the W Domain Repeat family (12, 43, and 75) which process rRNAs and are required for cell proliferation [31]. Enhanced ribosome biogenesis is associated with cell replication, notably the progression into S phase of the cell cycle [32,33]. Fitting with this, the most significantly enriched protein in unfixed hPACs, TNFRSF11B (Osteoprotegerin; Padj. ​= ​4.5 ​× ​10^−4^, log_2_FC ​= ​4.00 [3.74–4.26]) has known roles in driving chondrocyte proliferation and hypertrophic differentiation [[34], [35], [36]].

Thirty-three enriched proteins mapped to cholesterol and steroid biosynthesis pathways (Table S6). Of note, HMGCR (3-hydroxy-3-methylglutaryl-coenzyme A reductase) was uniquely detected in the unfixed hPACs (Fig. 1B). Other proteins mapping to these pathways included CYP51A1 (Lanosterol 14-alpha demethylase; Padj. ​= ​0.003, log_2_FC ​= ​2.19 [1.93–2.44]), FDFT1 (Squalene synthase; Padj. ​= ​0.004, log_2_FC ​= ​2.56[2.20–2.91]), and HMGCS1 (Hydroxymethylglutaryl-CoA synthase, cytoplasmic; Padj. ​= ​0.003, log_2_FC ​= ​2.90 [2.57–3.27]) (Fig. 2C), all of which were amongst the ten most significantly enriched proteins (Table S5).

In the unfixed cells, we detected increases of the proteins HMOX1 (Heme oxygenase 1, HO-1) and NOS2 (Nitric oxide synthase-2, NOS-2) in response to the collagenase treatment (unfixed cells). HO-1, the rate limiting enzyme in the catabolism of heme [37], is upregulated in a cytoprotective response to cellular stress [38]. HO-1 had the highest FC of all proteins detected between the two conditions (FDR ​= ​0.007, log_2_FC ​= ​7.16 [5.80–8.52]; Table S5). NOS-2 was uniquely, yet robustly, detected in the unfixed chondrocytes with an intensity rank of 2174 (Fig. 2B). There is an established link between the expression of nitric oxide and the induction of HO-1 expression in chondrocytes [39].

### 3.3 Transcriptomic shift between ground tissue and isolated cells

**Fig. 4:**
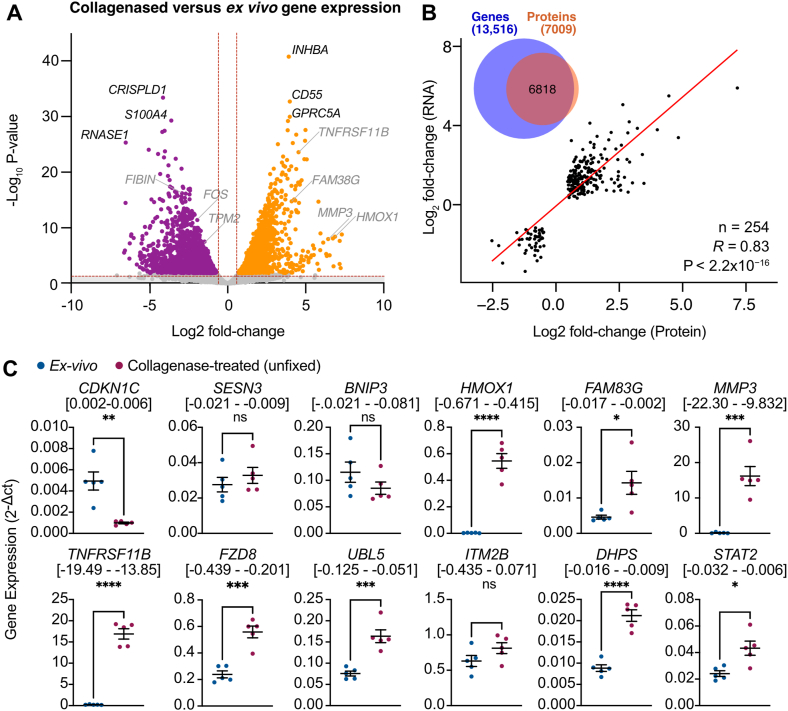
**Transcriptomic shift during collagenase treatment of human primary chondrocytes. A,** Volcano plot of the DEG following 18h collagenase digest of chondrocytes compared to *ex vivo* tissue. Purple, significantly decreased (BH-adjusted P ​< ​0.05, log_2_FC ​< ​−1.0); orange, significantly increased (BH-adjusted P ​< ​0.05, log_2_FC ​> ​1.0); grey, not significant. **B,** Correlation of effect sizes of genes and proteins which significantly change in the same direction across both datasets (Padj. <0.05). Venn diagram shows the overlap between the measured genes and proteins. **C,** Gene expression of 12 genes which were significantly differentially expressed in both the transcriptomic and proteomic data measured by qPCR in *ex vivo* (ground cartilage, n ​= ​5) RNA or that from unfixed collagenase-treated chondrocytes (n ​= ​5). ∗∗∗∗, P ​< ​0.0001; ∗∗∗, P ​< ​0.001; ∗∗, P ​< ​0.01; ∗, P ​< ​0.05; ns, P ​> ​0.05 calculated using an unpaired *t*-test. (For interpretation of the references to color in this figure legend, the reader is referred to the Web version of this article.)

To compare the proteomics data with transcriptomic changes, we next re-analysed an RNA-seq dataset which had been previously generated to compare the transcriptomes of ground cartilage (*ex vivo*) and hPACs isolated via an 18h collagenase digest protocol which is detailed in the original publication [11]. The cell isolation methodology differed to ours in the duration, type of collagenase used, and the use of hypoxic conditions. This dataset (GSE217871) was originally generated to optimize cartilage digest conditions, yet specific transcriptomic changes between the samples were not reported. We chose to reanalyze and integrate this dataset to validate our findings and test for robust changes which occur at the gene/protein level during hPAC isolation independent of the exact methodology. We identified 3937 differentially expressed genes (Padj. < 0.05, log_2_FC ​> ​1.0; Table S8). Of these, 2034 were significantly higher in the hPACs than in the *ex vivo* RNA (Padj. ​= ​0.049–2.95 ​× ​10^−37^, log_2_FC ​= ​1.00–7.32) and 1903 significantly lower (P = 0.049 - 3,39×10^−30^ log_2_FC ​= ​−1.00 to −6.62; Fig. 4A).

Of the 13,516 detected genes, 6818 of the encoded proteins were also present in our proteomic dataset (Table S9), representing 97.3 ​% of the hPAC proteome (Fig. 4B). Based on BH-adjusted P-values, there was an overlap of 304 differentially expressed genes (DEG) and proteins (DEP) between the two datasets, 276 of which (including 22/24 unique proteins) changed in a concordant direction (91 ​%), with a significant correlation in effect sizes (R ​= ​0.83, P ​< ​2.2 ​× ​10^−16^; Fig. 4B). Twenty-eight genes/proteins were differentially expressed in opposing directions (Table S10; Fig. S9).

To validate the observed results, we isolated mRNA from an independent cohort of patient samples from ground cartilage (*ex vivo*), or through collagenase digest of unfixed tissue (both n ​= ​5). Using qPCR, we first measured the expression of seven genes which were concordantly differentially expressed in our proteomic and transcriptomic analyses: *CDKN1C, SESN3,* and *BNIP3*, which were unique to the fixed chondrocytes in the proteomic dataset, and significantly decreased (Padj. ​= ​0.009 [−2.9 to −0.7], 3.4 ​× ​10^−5^ [−3.5 to −1.5], 2.8 ​× ​10^−5^ [−2.2 to −0.9], respectively) in the transcriptomic dataset; and *HMOX1, FAM83G, MMP3,* and *TNFRSF11B,* which significantly increased in both the proteomic (Padj. ​= ​0.01 [3.6–6.1], 0.02 [2.8–6.1], 4.5 ​× ​10^−4^ [3.7–4.3], respectively) and transcriptomic datasets (Padj. ​= ​2.6 ​× ​10^−13^ [3.1–5.1], 3.3 ​× ​10^−7^ [4.2–8.6], 2.2 ​× ​10^−21^ [3.7–5.4], respectively). Five of the genes also showed decreases in expression by qPCR (Fig. 4C), whereas *SESN3* and *BNIP3* did not show significant gene expression changes (P ​= ​0.42 and 0.21, respectively).

We next measured the expression of five genes which were differentially expressed in both datasets, however in a discordant manner. *FZD8, UBL5,* and *ITM2B* significantly decreased in the transcriptomic dataset (Padj. ​= ​1.5 ​× ​10^−4^ [−3.4 to −1.3], 0.003 [−1.6 to −0.47], and 0.04 [−1.3 to −0.2], respectively) and increased in our proteomic study (Padj. ​= ​0.017 [1.8–3.2], 0.015 [0.8–1.5], and 0.015 [0.7–1.3], respectively). By RT-qPCR, we identified increases in expression of *FZD8* (P ​= ​3.0 ​× ​10^−4^ [0.2–0.4]), *UBL5* (P ​= ​6.0 ​× ​10^−4^, [0.05–0.13]), and *ITM2B* (P ​= ​0.136 [−0.1 – 0.4]; Fig. 4D), following collagenase treatment, consistent with the results of our proteomic analysis. For visualization, the normalized values gene expression values are displayed along with the normalized protein and transcript expression levels (Table S11) in Supplementary Fig. 10.

Conversely, *DHPS* and *STAT2*, significantly decreased in the proteomic dataset (Padj. ​= ​0.04 [−2.0 to −0.6] and 0.004 [−1.7 to −1.1], respectively) and increased in the transcriptomic analysis (Padj. ​= ​0.001 [0.6–2.4] and 6.2 ​× ​10^−4^ [0.5–1.8], respectively). RT-qPCR revealed significant increases of both *DHPS* (P ​= ​4.9 ​× ​10^−5^ [0.01–0.02]) and *STAT2* (P ​= ​0.011 [0.01–0.03]), consistent with the transcriptomic dataset (Fig. S10).

### 3.4 No observable shift in cell cycle was detected following collagenase treatment

At the proteome level, we observed an increase in proteins mapping to ribosomal biogenesis pathways, potentially indicative of a shift in the cell cycle. To test this, we repeated our fixation and digest protocol on cartilage from three additional donors. Following digestion (with or without prior fixation), cells were stained with propidium iodide, and the cell cycle stage was quantified by flow cytometry (Fig. S12).

**Fig. 5:**
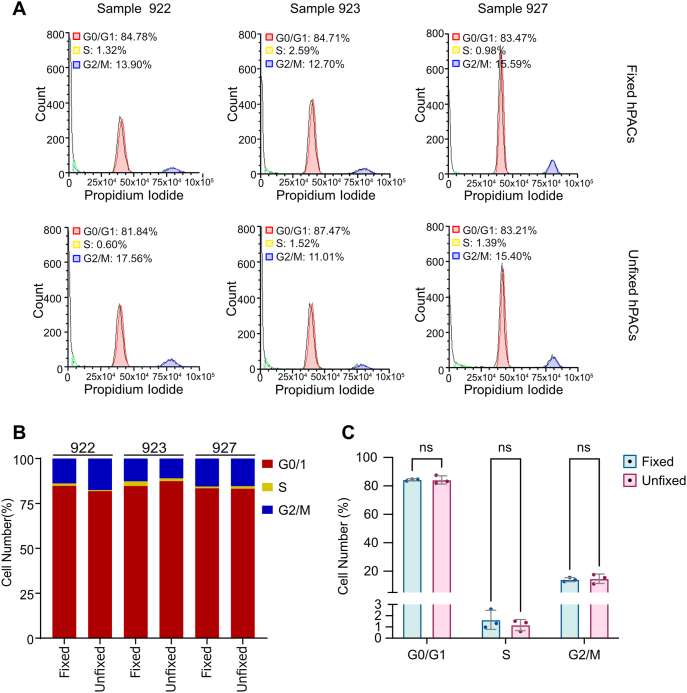
**Cell cycle analysis in fixed and unfixed hPACs. A,** Cell cycle profile of fixed and unfixed hPACs from three independent donors. Red, G0/1; yellow, S; blue, G2/M. **B,** Proportions of cells in each cell cycle phase. **C,** Bar plot of the percentage of fixed (blue) and unfixed (pink) cells within each cell cycle phase. Each point represents an individual donor. Horizontal bars represent the mean ​± ​SEM. ns, P ​> ​0.05 calculated using multiple paired t-tests and Benjamini-Hochberg FDR correction. (For interpretation of the references to color in this figure legend, the reader is referred to the Web version of this article.)

Across both conditions, the majority of cells (81.8–87.5 ​%) were in G0/G1 phase of the cell cycle, with 11.0–17.6 ​% in G2/M and <2.6 ​% in S Phase (Fig. 5), indicating that most cells were not actively dividing. No significant difference was identified between the fixed and unfixed hPACs when comparing the proportions of cells in each of the phases (FDR >0.05; Fig. 5).

## 4 Discussion

The primary aims of this study were to present the proteomic fingerprint of the primary human chondrocyte in OA, and to fully characterize the proteomic shift in hPAC phenotype following collagenase digest. We believe that this dataset will serve as a valuable resource to the osteoarthritis research community by increasing our understanding of how the cells change during isolation processes. The proteome of the fixed chondrocyte presented here may further serve as a reference for the *ex vivo* phenotype of the OA hPAC and inform researchers seeking to modulate specific genes and pathways in primary cells.

We identified a non-significant 10 ​% increase in cellular protein mass in hPACs following collagenase treatment. This was confirmed by an increase in the estimated copy numbers of proteins per chondrocyte, which appeared to be consistent across all protein categories investigated. We further tested for differentially expressed proteins (DEP) between the fixed and unfixed hPACs, identifying a significant downregulation of the transcription factors FoxO1 and FoxO3. The FoxO signaling pathway is a known regulator of cell proliferation, survival, and nutrient metabolism and plays a chondroprotective role in the joint through regulating the expression of matrix genes [29]. Notably, FoxO1 has been shown to induce cell cycle arrest in the G0/G1 phase in chondrocytes [40]. Other cell cycle inhibitors including BNIP3 and CKDN1C were uniquely detected in the fixed hPACs, with their expression dropping below the detectable limit in the unfixed cells following collagenase treatment. Fitting with this observation, the most highly enriched pathways in the unfixed hPAC proteomes were associated with ribosome biogenesis, a process typically associated with cell proliferation [41]. Our investigations using propidium iodide did not identify a shift in the cell cycle between the fixed and unfixed hPACs. The majority of the cells remained in a non-proliferative state, comparable with previously published results, which reported 83 ​% of isolated hPACs in G0/1 [27]. However, our sample size was modest (n ​= ​3) and, in primary cells, the S phase can be very quick meaning that we may require more power to detect a true effect. Propidium iodide staining provides a sensitive and robust assay to determine cell cycle state and is widely employed [42]. However, this assay does not specifically distinguish between cells in very early or late S phase, from cells in G1 and G2 phase respectively. To investigate this, an alternative technique, such as bromodeoxyuridine (BrdU), would be required and warrants further investigation. Alternatively, other processes are linked to increased ribosome biogenesis including cellular differentiation. However, the only relevant enriched pathway term in the unfixed hPACs was “osteoclast differentiation” driven by the upregulation of proteins including FOSL1/2, JAK, and TGFB1, which has a multitude of known roles in chondrocyte biology.

The transcriptome acts as a direct link between the genome and proteome and, consequently, a common misconception is that relationships across biological scales are proportional. Yet, the relationship between mRNA and protein levels is not always straightforward due to an extensive array of post-transcriptional and post-translational modifications impacting stability [43] and the higher dynamic range of protein abundances compared to transcript [44]. Most studies directly investigating this relationship have investigated paired datasets to test the correlation between the transcriptome and proteome [45,46], rather overlapping differential expression profiles, as we have tested here. In this study we overlapped DEP and DEG between two independent datasets which had been generated on the same cell type, using comparable (but not identical) methodologies. Overall, 53 ​% of our DEP were also seen to significantly change at the transcriptomic level. Considering the differences in samples used (and modest sample number in both studies), methodology, and research groups conducting the studies, this overlap is not inconsiderable and validates that those genes/proteins which were consistently identified represent a true phenotypic shift during hPAC isolation. Furthermore, the DEP/DEG changed with comparable effect sizes, and >90 ​% were concordant in direction. This further indicates that these molecules can be expected to change in expression regardless of the exact method of hPAC isolation, which can vary considerably between laboratories. The extent of this overlap may also be limited by the use of DESeq2 to determine significant expression changes, which is designed to be applied to datasets of larger sample numbers. Surprisingly, almost 10 ​% of differential expression was discordant between the datasets and we sought to investigate this further. Our RT-qPCR analysis identified that this could not solely be attributed to differences in methodologies. Of the five genes which we followed up, three increased following collagenase treatment (*FZD8, UBL5, ITM2B*), consistent with our proteomic data. This indicates that the increase could potentially be due to the exposure of the hPACs to a normoxic environment as the transcriptomic dataset was generated under hypoxic conditions. However, *DHPS* and *STAT2* both decreased at the protein level following collagenase treatment, yet increased at a transcriptomic level, which was confirmed by our qPCR. This highlights the complexity of the transcriptome-proteome relationship and cautions against the heavy reliance upon transcriptomic data alone to elucidate biology. The community has leaned heavily into transcriptomic data over the last two decades, first through microarrays and more recently through next-generation sequencing approaches [47]. Whilst this is largely due to accessibility and economic factors, it would be prudent to functionally validate many “hits” in transcriptomic studies to be able to draw robust biological conclusions. That said, a limitation of this study, is that it is not possible to entirely distinguish between “false discordance”, a result of methodological variation, and “true discordance” where an uncaptured level of biological variation i.e., miRNAs or post-translational regulation, may be driving a real paradoxical effect [48].

A limitation of this study is that we have not deconvoluted the impact of the normoxic environment upon hPACs from the stress response to collagenase digest and loss of matrix sensing. However, many laboratories do not choose to routinely culture hPACs in hypoxic environments. Here we have demonstrated that the enzymatic isolation of the cells in normoxic conditions has a substantial impact on the cellular proteomic fingerprint, yet, overall, the hPACs retain their chondrocyte phenotype post-digest. It would be of future interest to determine whether advanced culture techniques such as hypoxia (1 ​% O_2_) or 3D culture models could shift the phenotype back to that of the *ex vivo* chondrocyte, captured here in the fixed hPAC populations. A strength of the dataset described herein is that we present a reference of the *ex vivo* chondrocyte proteome against which such interventions could be benchmarked.

In conclusion, we have presented the proteomic fingerprint of the *ex vivo* human primary chondrocyte from an OA knee joint. We have reported specific change in genes and pathways across multiple scales of biology, which in many cases remain consistent despite the specific methodology used. We believe that this dataset and associated analyses can serve as a valuable reference for the community and encourage the use of tissue fixation or alternative methods to preserve the multiome where permissible for downstream applications.

## Data availability

The mass spectrometry proteomics data have been deposited to the ProteomeXchange Consortium via the PRIDE partner repository [49] with the dataset identifier PXD064948. Reviewers can access the data through the following details: Project accession: PXD064948; Token: BarcyaysuXdS. All other data and analysis code is available from the corresponding authors upon request.

## Author contributions

SJR, MT, NJC and DJD supervised the study, interpreted the data, and procured funding. AB, SR, WC, and CT performed experiments. EBJ and SEO performed bioinformatic analysis. All authors contributed to scientific discussions and the preparation of the manuscript.

## Funding

This project was funded by a Versus Arthritis Career Development Fellowship awarded to SJR (22615), the EPSRC MoSMed Centre for Doctoral Training, and Nuchido Ltd. CT is funded by the Newcastle upon Tyne Hospitals Charity TEAM study grant awarded to DJD.

## Declaration of competing interest

Author NJC is Founder and Chief Executive Officer of Nuchido Ltd. and was involved in the study design and manuscript preparation.
