# Supplementary material for: A proteomics investigation of primary human articular chondrocyte isolation
Source: Osteoarthr Cartil Open. 2025 Aug 23;7(4):100664. doi: 10.1016/j.ocarto.2025.100664 (PMC12506514; doi:10.1016/j.ocarto.2025.100664)
Supplement: Multimedia component 1 [file mmc1.docx]

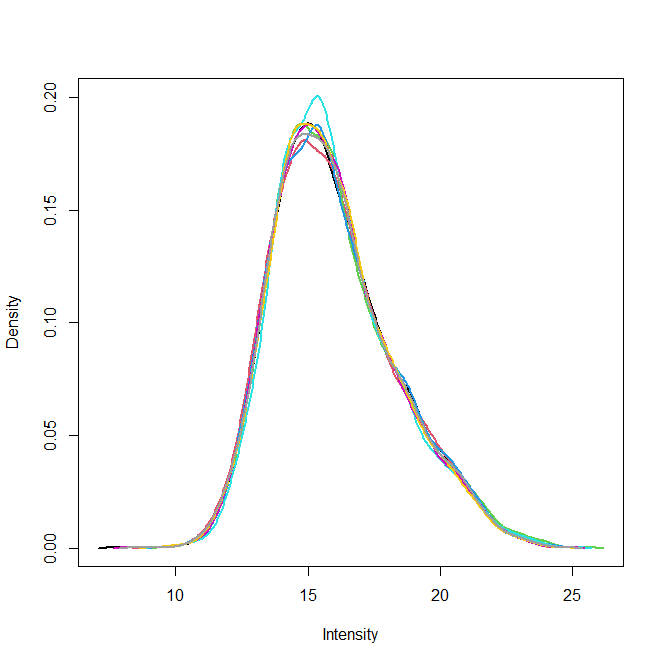


**Supplementary Figure 1.** Distribution of log_2_ label-free quantification (LFQ) intensities across samples.


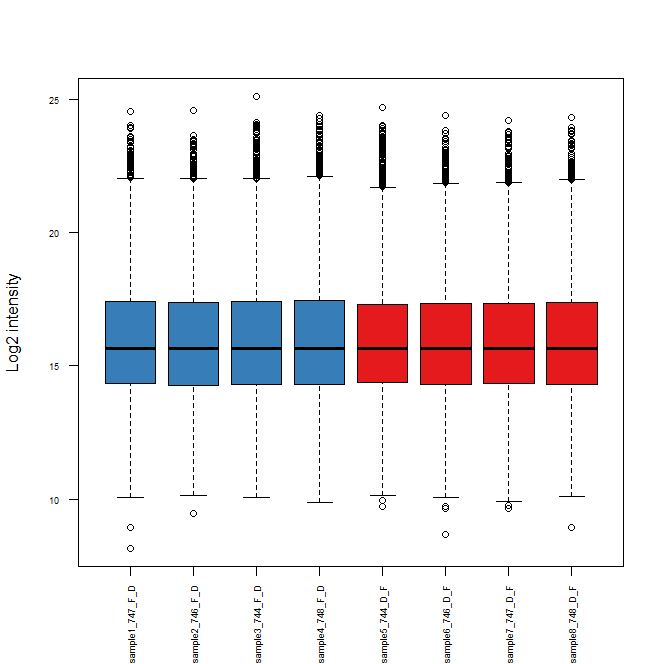


**Supplementary Figure 2.** Boxplot of the log_2_ label-free quantification (LFQ) intensities across samples.

**Supplementary Figure 3.** Plot showing dispersion estimation of the mean of the normalised counts. The mean of the normalised counts and the likely estimated dispersion based on read counts (blue dots), compared to the expected dispersion value for genes of a given expression strength (red line).

**Supplmentary Figure 4.** Heatmap showing clustering of log transformed DeSeq Results comparing cartilage tissue collagenase-treated for 18hours with 0.1% collagenase II and *Ex Vivo* ground cartilage tissue.

**
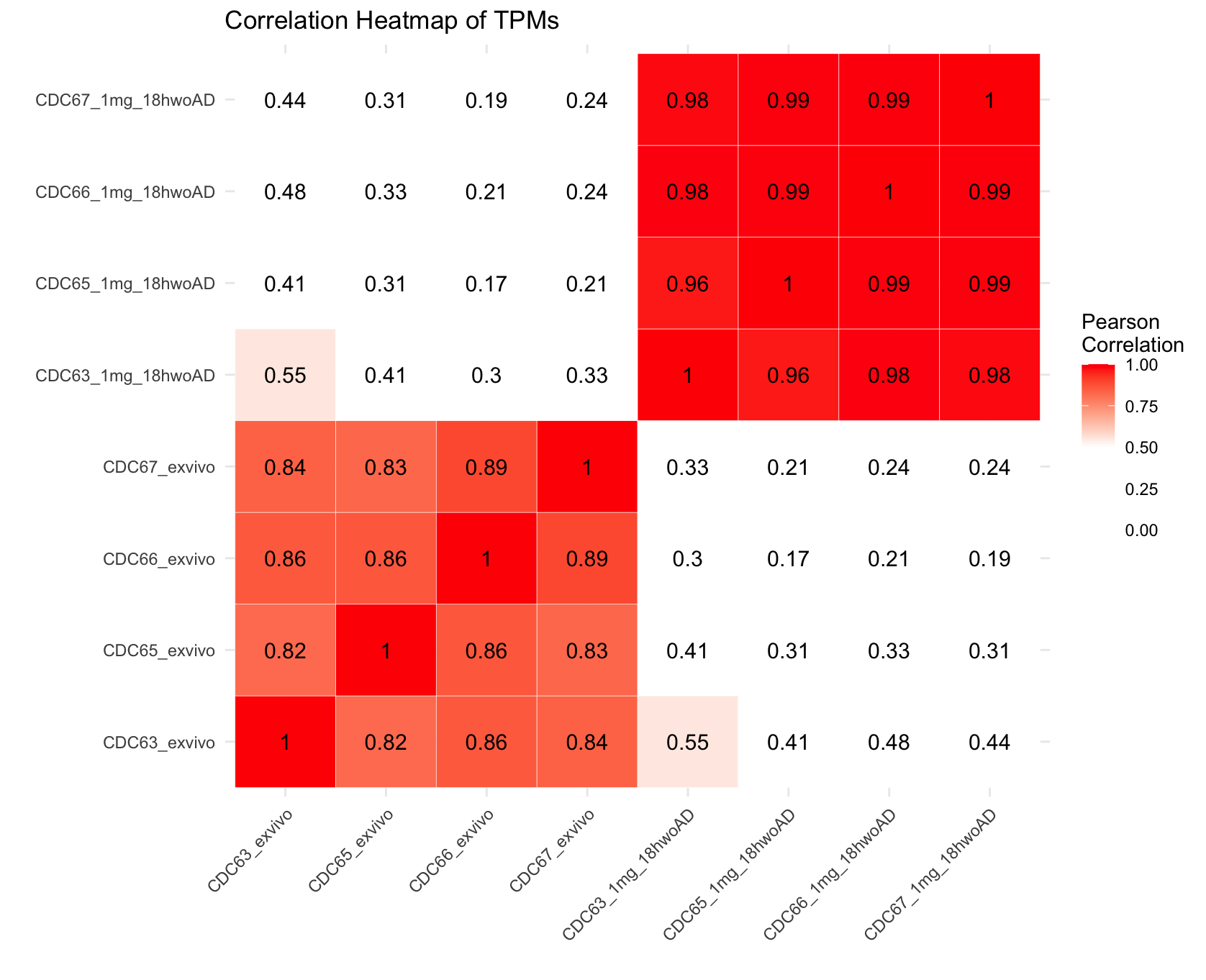
**

**Supplementary Figure 5.** Heatmap showing Pearson correlation coefficients between all pairs of samples based on TPM values. Warmer color (red) indicates stronger correlations.

**Supplementary Figure 6.** Density plots of log-transformed TPM values (log₁₀(TPM + 1)) for all samples. Each curve represents one sample, with TPM distributions derived from RNA-seq gene-level expression. The overall shape reflects the typical right-skewed expression distribution, with most genes expressed at low levels and a smaller number highly expressed.

**Supplementary Figure 7.** Bar plot of estimated protein copies per cell in each of the 14 assigned biological categories. Blue, fixed; pink, unfixed hPACs.

**Supplementary Figure 8.** Full STRING network of 38 protein network mapping to ribosome biogenesis pathways in our curated pathway analysis. The thickness of the edges (dark blue) indicates the confidence of interaction based on experimental data and database evidence.

**Supplementary Figure 9.** Plot comparing the effect sizes of differentially expressed RNA and protein in fixed and unfixed cartilage tissue.

**Supplementary Figure 10.** Normalised expression of unfixed (pink) hPAC proteins (measured by LC-MS/MS), transcripts (measured by RNA-seq) and genes (measured by qPCR). Protein levels are normalised to the fixed hPAC condition. Transcript and gene expression is normalised to the ground cartilage (*ex vivo*) control (both shown in blue). These plots are to allow visualisation of the comparative changes in expression measured through the three methods.

**
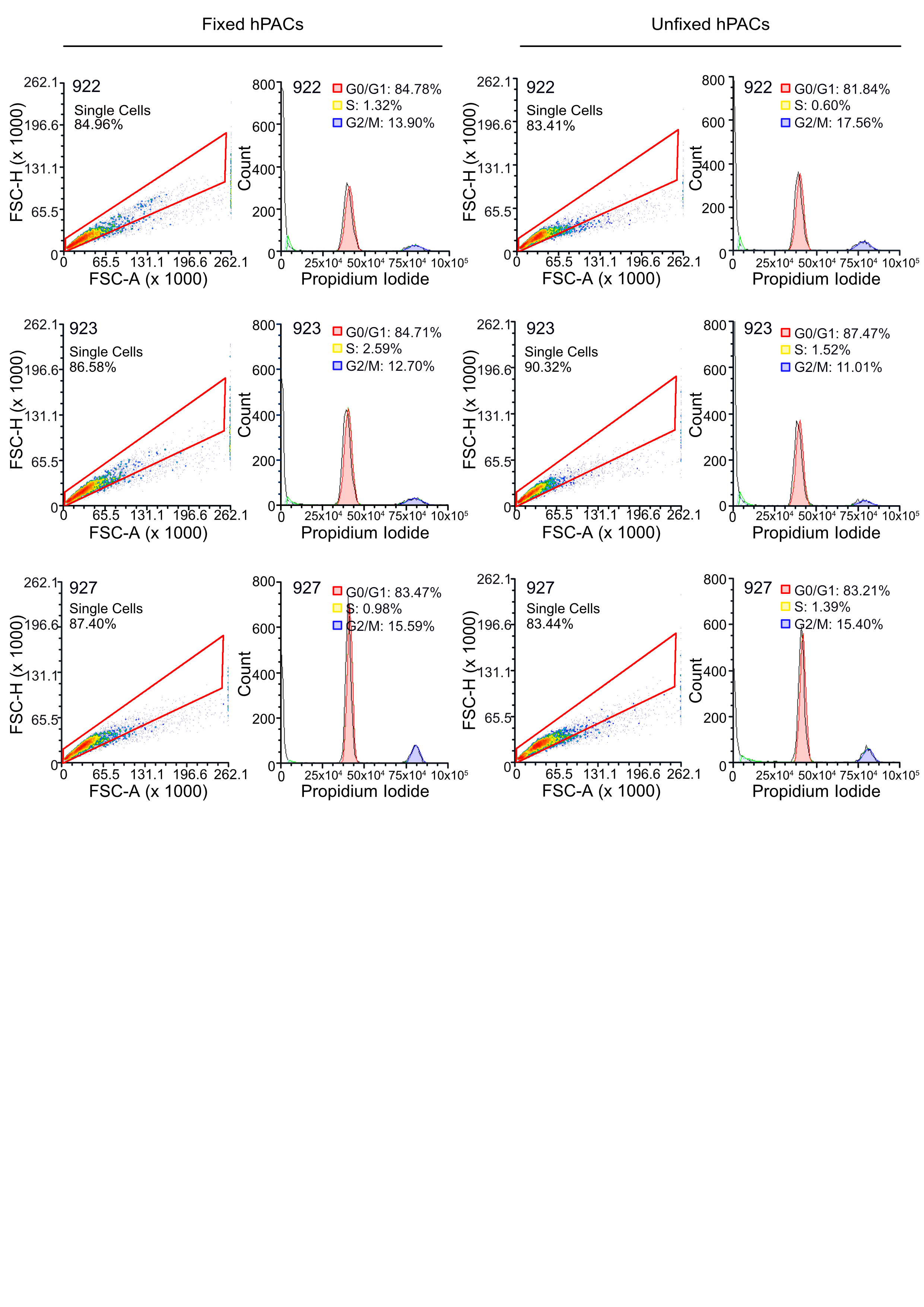
**

**Supplementary Figure 11.** Analysis of cell cycle in fixed (left) and unfixed (right) hPACs (n=3) by propidium iodide staining and flow cytometry. LHS, gating to exclude doublets and debris based on FSC-H vs FSC-A. RHS, Cell cycle profile of fixed and unfixed hPACs from three independent donors. Red, G0/1; yellow, S; blue, G2/M.
